# Using Metal Magnetic Memory to Evaluate the Effect of Welding Method and Weld Temperature on Magnetic Field Strength in Structural Steel

**DOI:** 10.3390/ma16155256

**Published:** 2023-07-26

**Authors:** Agata Wieczorska, Agnieszka Kosoń-Schab

**Affiliations:** 1 Faculty of Marine Engineering, Gdynia Maritime University, Morska St. 81–87, 81-225 Gdynia, Poland; a.wieczorska@wm.umg.edu.pl; 2 Faculty of Mechanical Engineering and Robotics, AGH University of Science and Technology, A. Mickiewicza Av. 30, 30-059 Krakow, Poland

**Keywords:** metal magnetic memory, magnetic field non-destructive evaluation

## Abstract

Tests of welds are carried out inter-operatively, during the execution of the steel structure, as well as after the structure is welded, but even before its assembly. Steel structures already in service are also examined to detect potential cracks, delamination, or corrosion loss of thickness having the effect of weakening the mechanical strength of the structure. Such examinations are some the elements that comprise a structural health assessment. In this paper, the metal memory method was used to evaluate the effect of the welding method and weld temperature on the change in magnetic field strength. S235JR structural steel was used for the study, which was subjected to milling and MMA, TIG, and MIG welding. The results of measurement experiments carried out for each welding method are presented as graphs.

## 1. Introduction

To realize reliable action and proper operation of machines and their structures, information on the condition of the material must be obtained. Ideally, this information should be obtained by non-invasive means. Therefore, the method of magnetic metal memory (MMM) is increasingly being used to predict and prevent failure [1,2]. The method is based on the use of the phenomenon of magnetic memory of the metal, which involves changing the magnetic properties of the material resulting from the presence of defects and structural damage in it. MMM is a passive non-destructive magnetic method using self-magnetic leakage field distribution (SMLF) on the surface of materials. The variation of the SMFL signal is related to many factors, such as the initial magnetic field, microstructure, chemical composition, and shape and size of ferromagnetic materials [3,4,5,6]. The value measured in the test is the value of the selected component of the magnetic field strength H measured near the diagnosed object, represented by Formula (1).

(1) $$H=\frac{\beta }{\mu_{r}*\mu_{0}}$$

where [5]: *H*—magnetic field strength [A/m], *β*—magnetic induction [T], *µ_r_*—relative magnetic permeability, *µ*_0_ is vacuum magnetic permeability 4 π × 10 − 7 [H/m].

Information on magnetic field strength is determined by the tangential and normal components of MMM signals. If these components reach a maximum value and pass through zero, there is a change in polarity. Then, in the areas of stress concentration zones (SCZs), we speak of magnetic anomalies indicating a potential damage site [7]. It has been proven in many works that MMM output signals have a good correlation with stress anomalies on the surface of ferromagnetic materials [8,9].

Previous studies have indicated that residual stresses and stress concentrations can have an impact on mechanical properties, erosion resistance, dimensional precision, fatigue damage resistance, and structural life [8,10]. In papers [11,12], it was proven that residual stress affects the magnetic properties of ferromagnetic materials. Initial residual stress states have been shown to have a potent influence on normal component (Hy) signal changes caused by tensile stresses, especially in the elastic phase. In contrast, these states do not significantly affect the tangential component (Hx) signal. The magnetic signal decreases or increases the residual magnetization. For an unmagnetized sample, it always tends toward the residual magnetization and approaches the stable one, which can be well explained by a magnetomechanical model based on the “effective field theory” and “law of approach”. The increase in applied stress affects the magnetic signal for the demagnetized sample. It fluctuates slightly around the initial value of the magnetic field. A manuscript developed by Leng et al. discussed possible reasons for this behavior underlying magnetic trends and proposed an accurate physical model [13]. Concerned for the safety of people using equipment based on structures made of ferromagnetic materials, a method and test system have been developed to study the effect of steel mass on magnetic field distribution [14].

The MMM method is often used for qualitative evaluation of welded joints. Unlike other defectoscopic methods, MMM diagnostics do not require preparation and pretreatment. Dirt, a layer of varnish or grease has no effect on the measurement result [15,16]. At the production stage, cleaning of the surface after welding is required. At the operation stage, the removal of loose deposits from the joint surface is required. These joints, after nondestructive testing at the production stage, are not free of defects. These defects, along with imperfections at the micro level, concentrate stresses from working loads. By finding zones of stress concentration, areas of potential cracking can be found. The effect of the thickness of the corroded layer or other joint coatings on the test results should be taken into account, and sensitivity tests for detecting indications should be carried out. External magnetic fields, such as operating electrical equipment, magnetization during magnetic-powder testing, or current flow near the test piece during welding, may limit the use and reliability of the method’s indications [17,18]. It has been investigated that the welded joint is a stress concentrator. In welded austenitic steels, inhomogeneity of delta ferrite in the weld causes changes in residual magnetic field (RMF) distribution and gives indications.

Knowing the potential of the MMM test method, it seems reasonable that it should be used in the in-service testing of welded joints.

Defects in welded joints are potential stress concentrators. However, stress concentration in a welded joint is not necessarily the result of defects alone. Looking more broadly at the problem of stress concentration, a welded joint, due to the notch effect and thermal deformation after welding, is itself a stress concentrator. Therefore, not all indications obtained by MMM testing are defects in the colloquial, standard sense. Some indications of the MMM method for welded joints may be due to the presence of an uneven distribution of tension after welding, which may be related to the cooling rate of the weld. Using the Magnetic Barkhausen Noise (MBN) technique in conjunction with the MMM technique, the stresses in a welded steel ship sheet were investigated. As part of this, a three-dimensional image of the magnetic field of the spontaneous leakage was obtained. In addition, MBN measurements were taken in the same test area. It was found that the stress distribution deduced from the MBN measurements corresponded well with the MMM self-sealing image. As a consequence of this positive result, a new test procedure using a combined MBN and MMM technique was proposed [19].

To apply metallic magnetic memory technology to welding residual stress testing, a hardware and software MMM system has been designed. The software subsystem is based on object-oriented programming, and the hardware subsystem is portable. The MMM system can study the distribution of residual stress in a welded joint, residual stress concentration zones, and defects in a shop [20].

The ferromagnetic structure contains defects, and the defect size and morphology must be accurately reported to confirm whether the structure is safe. A fundamental problem in quantitative MMM studies is the quantitative analysis of defect location and size based on MMM signals [18].

It has been shown that it is possible to evaluate defects in welded joints quantitatively by examining the MMM signal using a welded joint sample from Q235 steel [21].

Dubov and Kolokolnikov conducted a comprehensive MMM study of the base metal and welded joints of steel structures and parts of steam turbines, pipelines, and steam tanks. According to the authors, the metal’s magnetic memory is a side effect. It is formed during their manufacture and cooling in the weak magnetic field of the earth. It occurs in the form of residual magnetization of the metal in components and welded joints. Using the method of magnetic memory of metal and other conventional methods of nondestructive testing and microstructure analysis, it has been shown that zones of stress concentration are sites of defects or degradation of material properties [15,22]. Compared with the small-hole stress testing method, Li et al. [11] showed that magnetic field anomalies near the surface of ferromagnetic material can be used to control residual stresses in the weld seam in a pipe specimen. They conducted fatigue tests for welded and unwelded Q345B and Q345qC specimens and investigated the MMM signal. Qi et al. [19] used the MMM technique to locate the region with high residual stresses and investigated the stresses in welded ship plates based on the magnetic Barkhausen Noise technique. Su et al. [23] measured the normal component of the MMM field for defective and non-defective butt-welded Q345 steel specimens.

In practice, a magnetic field is generated during welding. The strength of the magnetic field depends on the method and welding parameters, such as current intensity, type of electrode, and distance from the welding site. This is important when using welded parts near sensitive electronic equipment or magnetic components. In ferritic materials, the influence of an external magnetic field leads to magnetization of these materials. The magnetic field during welding can affect the performance of magnetic sensors, such as Hall sensors, proximity sensors that use magnetic fields to detect objects or determine distances. Strong magnetic fields can interfere with their operation or affect the accuracy of measurements. The purpose of the research is to determine the effect of the welding method on the change in SMLF of welded components in terms of analyzing the possibility of controlling their magnetization. An important part of the research is to obtain information about the changes in the self-magnetic leakage field in the welded component during the cooling of the weld.

## 2. Materials and Methods

Sheets of structural S235JR carbon steel measuring 70 × 150 × 5 mm were used for the study. These specimens were subjected to the welding process using 111 MMA (Shielded Metal Arc Welding, 111—Manual arc welding, with covered electrode), 135 MAG (Metal Active Gas, 135—Metal electrode welding with active gas shielding), and 141 TIG (Tungsten Inert Gas, 141—arc welding with tungsten electrode in an inert gas shield with solid wire/bar) methods. An invector welder Kraft&Dele KD811 40-400 A was used to make all the welds.

The welding process parameters were selected based on EN ISO 15614-1:2017-Specification and qualification of welding technologies for metallic materials [24]. The parameters are shown in Table 1. While Table 2 shows the chemical composition and mechanical properties of the filler metal [25,26].

**Figure 1:**
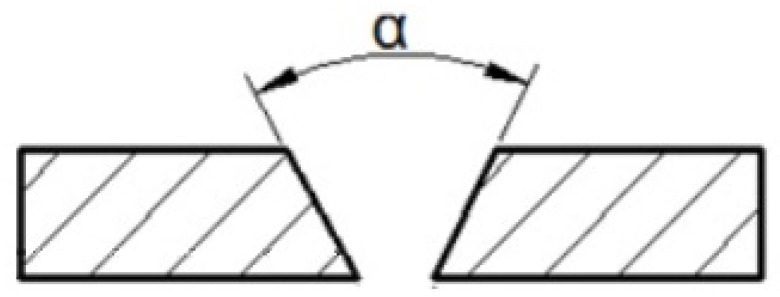
Beveled angle of face faces.

**Figure 2:**
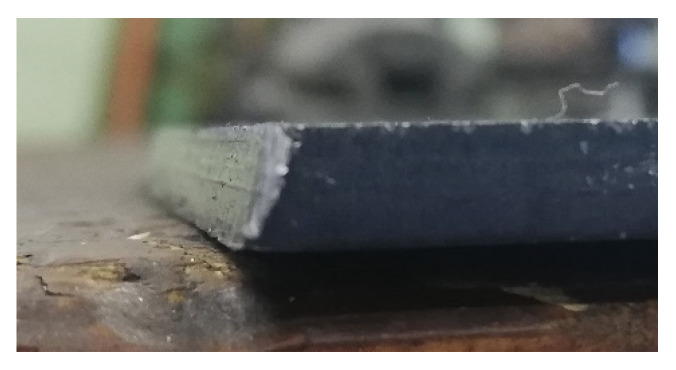
Steel plate after bevel milling.

The surfaces of the parts to be welded were cleaned by milling with the following parameters: 0.2 mm milling depth milling speed 500 rpm feed 20 mm/min. To obtain the required remelting and facilitate welding, milling of the faces at an angle of 25° was conducted on the samples to obtain an edge bevel by PN-EN ISO 9692-1:2014-02 [27] for welded parts up to 10 mm thick 40° < α < 60° (Figure 1 and Figure 2) [26]. The milled chamfer was made with an HS fi 16 burrs,(PAFANA brand) cutting speed 800 rpm, feed 20 mm/min, and water cooling.

**Figure 3:**
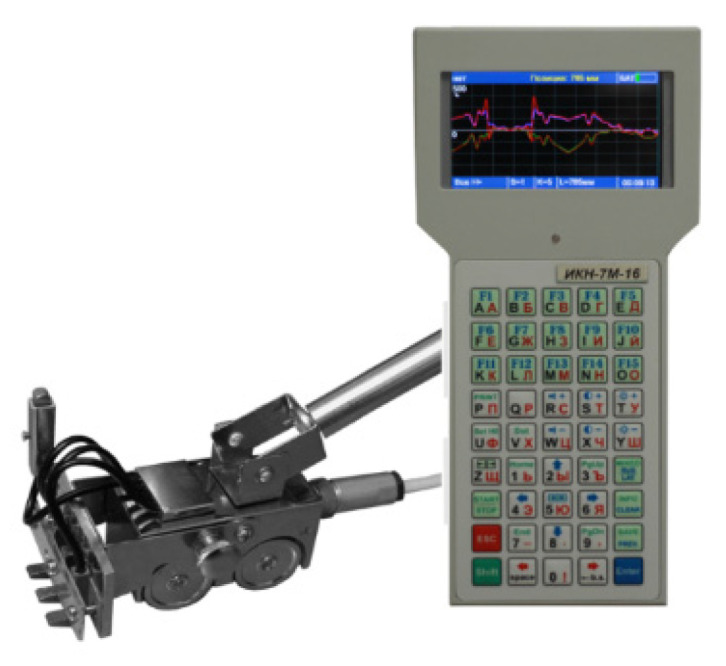
Tester of Stress Concentration TSC-4M-16 and scanning four-channel sensor.

A TSC-4M-16 gantry magnetometer (Tester Stress Concentration, manufactured by Energodiagnostika from Moscow, Russia) with a scanning device was used to measure the magnetic flux leakage (MFL) data of steel specimens. The scanning device consists of a four-wheel carriage with flux-gate transducers and an incremental encoder (Figure 3).

Transducers (magnetometers) installed in the scanning device allow measuring the 2D distribution of the SMFL signal along the surface of the structure under test. The TSC-4M-16 gantry magnetometer can measure magnetic field strength within ±2000 A/m range, has a 5% basic relative error for Hp, and a 0.9 mm shift in sensor accuracy. Data acquisition and analysis software are included with the TSC-4M-16 magnetometer. The sensor records changes in the magnetic field caused by structural damage, e.g., fatigue, cracks, corrosion, plastic deformation, etc.

Detection of these changes allows assessment of the structural state of the material and identification of areas susceptible to subsequent damage.

**Figure 4:**
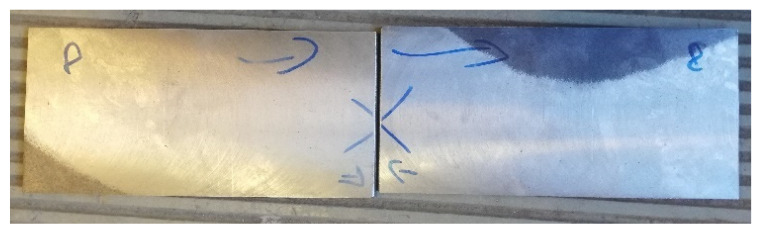
View of paired steel plates prepared for welding.

**Figure 5:**
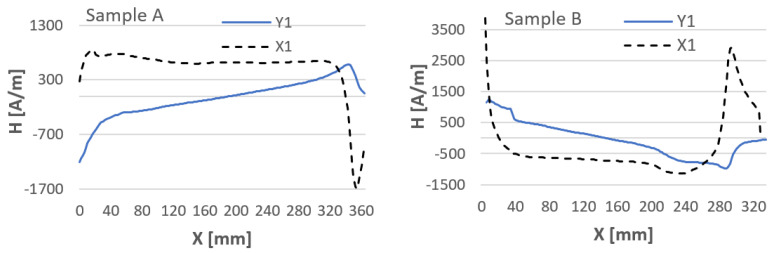
The magnetic field strength in plates A and B is not paired with each other.

**Figure 6:**
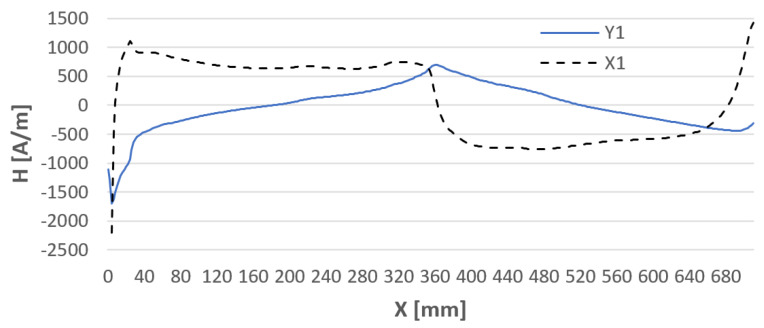
The magnetic field strength in samples A and B paired with each other.

Before testing the actual specimens prepared for testing, the steel sheets after the milling treatment were joined as they were to be joined together by welding (Figure 4). The connection was made to determine the magnetic field strength created when these samples were joined. An example of a graph of the magnetic field strength distribution before and after the joining of carbon structural steel sample specimens is shown in Figure 5 and Figure 6. The graphs of the components of the magnetic field intensity are described X1—the tangential component and Y1—the normal component for both samples.

**Figure 7:**
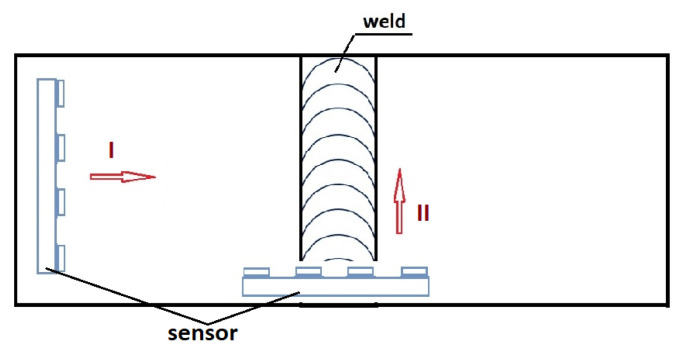
The direction of measurement of magnetic field strength distribution Hx and Hy for—I- plates before and after welding,—II- weld.

Analyzing the data after Hx and Hy measurements, it is noticeable that the steel samples are affected by each other’s magnetic field. When the sheets are joined, the magnetic fields of the samples interfere with each other, resulting in a change in H(x,y). Therefore, for research purposes, it is important to join the sheets and measure the state of their magnetic field strength before welding. To clarify how H(x,y) measurements are made, Figure 7 shows the initial points of application of the detector to the plates and their direction of measurement.

Measurement of the intensity of the H(x,y) value was made after each stage of the experiment. Each welding method was used to make three test specimens each. To avoid measurement errors, the magnetic field strength reading for each sample was repeated three times.

The first series of measurements were made after milling, cleaning and beveling the faces. In the next stage of testing, the samples were subjected to welding. Further measurements of the magnetic field strength H(x,y) were made after welding during the cooling of the weld.

**Figure 8:**
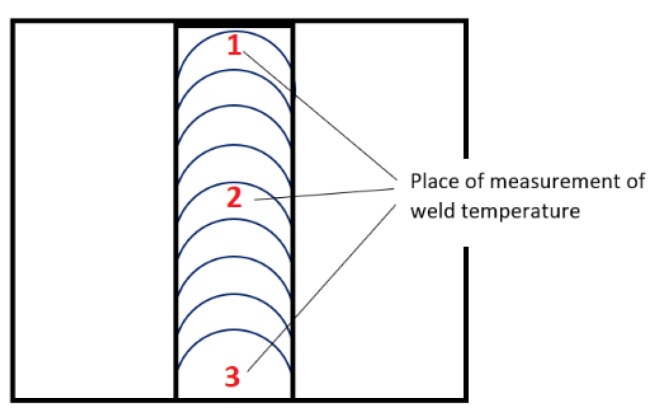
Temperature measurement in the weld.

Immediately after welding, the temperature was measured with an AB-8829 laser pyrometer (STANDARD INSTRUMENTS brand, Hong Kong, China), with a measuring range of −50 °C to 1000 °C. The measurement was performed by ISO 13916:2017 standard [28]. In each weld, temperature measurements were taken at three different locations: at both edges of the weld and in the middle of the weld, according to Figure 8. Table 3 shows the time and temperature measurement data during the cooling of the weld.

The time taken for the welds to cool down to ambient temperature (about 15 °C) was calculated. The magnetic field strength was measured at temperatures of 80 and 15 °C.

A visual examination of the weld was performed, which included the measurement of geometric dimensions using calipers and a weld gauge. With a magnifying glass, the weld was inspected for cracks, underfills, unfilled weld grooves, pores, overhangs, and leaks. Visual examination found no discrepancies. Weld quality acceptance level B was found. The tests were conducted by [PN-EN ISO 17637:2017, PN-EN ISO 5817] [29,30]. The sample was then subjected to penetration testing according to [PN-EN ISO 3452-1:2021, PN-EN ISO 5817] [31,32]. Using a Helling kit, a penetration test was conducted on the entire welding surface with a background illumination of 980lx. Through positive results on penetration testing, the fabricated welded joint also achieved weld class B.

## 3. Results

The results of magnetic field strength measurements were analyzed. During the analysis, the focus was on graphs based on the H-value for the entire connected part, and the weld itself. For a clear description of the graphs, the different stages of the tests, are marked: A—measurement of magnetic field strength before welding, B—after welding when the weld has a temperature of 80 °C, C—when the weld has a temperature of 15 °C. Welding methods are marked: 1-MMA, 2—TIG, 3—MAG. To make the graphs readable, only one example for each method was chosen. The components of the magnetic field strengths H(x) and H(y) are shown in separate graphs.

**Figure 9:**
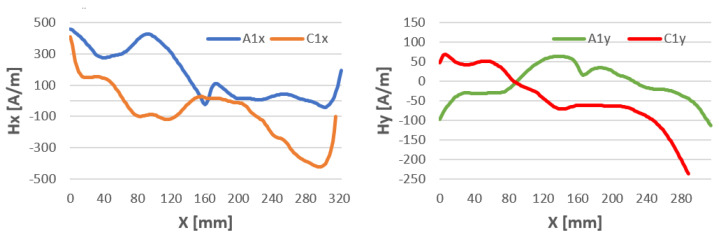
Magnetic field distribution Hx and Hy after milling—A and after welding by 111 MMA—C.

Samples welded using the 111 MMA method (Figure 9) show a completely different character of the magnetic intensity curve compared to the curve obtained after milling. There is a noticeable change in the polarity of the steels in the magnetic flux leakage after welding.

**Figure 10:**
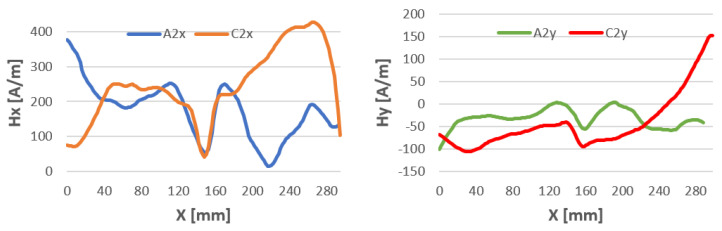
Magnetic field distribution Hx and Hy after milling—A and after welding by 141 TIG—C.

Samples welded by 141 TIG at the joining point have a pronounced magnetic anomaly, the same as for non-welded samples. The tangential component of Hx at the joining site for x in the 130–170 mm range takes the same value before and after welding (Figure 10). In contrast, the Hy component at this location is shifted by about 50 A/m.

**Figure 11:**
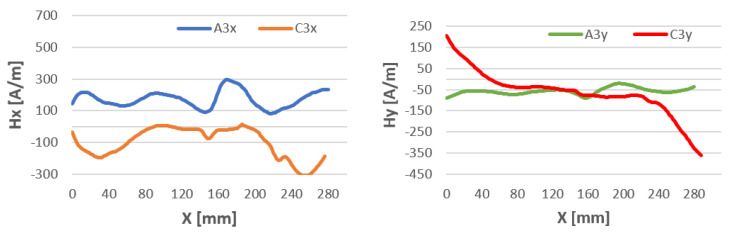
Magnetic field distribution Hx and Hy after milling—A, after welding by 135 MAG—C.

In 135 MAG welded specimens, the curve for tangential components maintains a similar course before welding. The values of Hx after welding are shifted below 0 compared to Hx before welding at the point of joining the material reaches values close to 0. The graphs of normal components cross, while at the point of joining they have the same values (Figure 11).

**Figure 12:**
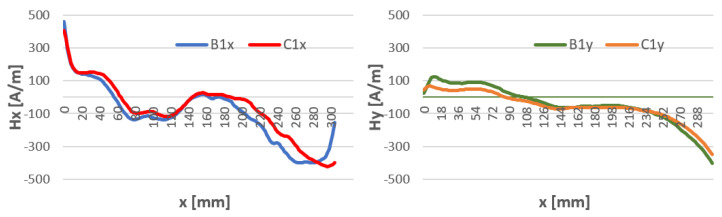
Comparison of the distribution of the magnetic field Hx and Hy of welded inserts for binders with temperatures of 80 °C (B) and 15 °C (C)—method 111 MMA.

**Figure 13:**
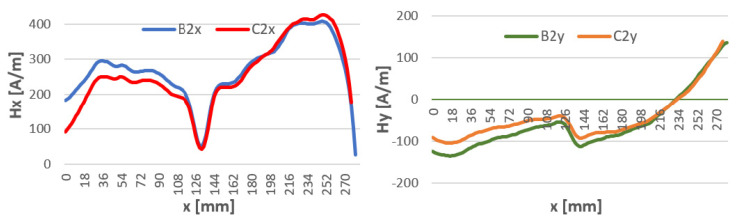
Comparison of the distribution of the magnetic field Hx and Hy of welded inserts for binders with temperatures of 80 °C (B) and 15 °C (C)—method 141 TIG.

**Figure 14:**
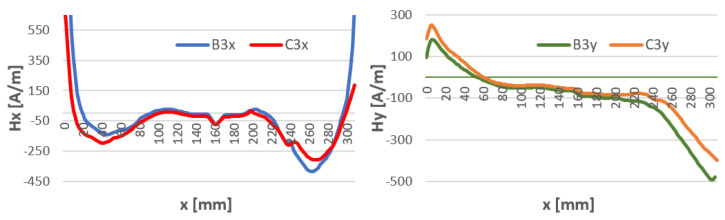
Comparison of the distribution of the magnetic field Hx and Hy of welded inserts for binders with temperatures of 80 °C (B) and 15 °C (C)—method 135 MAG.

To see if the time distance from welding and the temperature of the welding affects the magnetic field strength, H-charts were compared for temperatures: 5 min after welding where the weld reached a temperature of about 80 °C and at 15 min after welding for a weld temperature of about 15 °C. The waveforms along the length of the specimen are shown in Figure 9, Figure 10 and Figure 11. For all welding methods, the curves and Hx and Hy do not differ at the weld spot (x = 120–180) for all components. Outside the weld, the curves are shifted to each other. This may be due to a change in heat stress after the weld cools. Graphs of the magnetic field strength in the sample at intervals are shown in Figure 12, Figure 13 and Figure 14.

**Figure 15:**
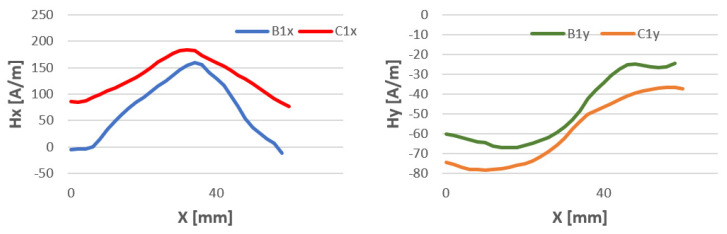
Comparison of the magnetic field distribution Hx and Hy of the binder at temperatures of 80 °C (B) and 15 °C (C)—method 111 MMA.

**Figure 16:**
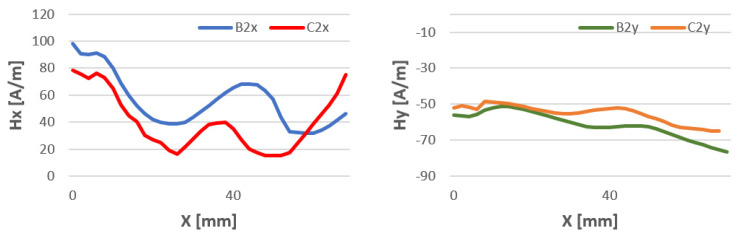
Comparison of the magnetic field distribution Hx and Hy of the binder at temperatures of 80 °C (B) and 15 °C (C)—method 141 TIG.

**Figure 17:**
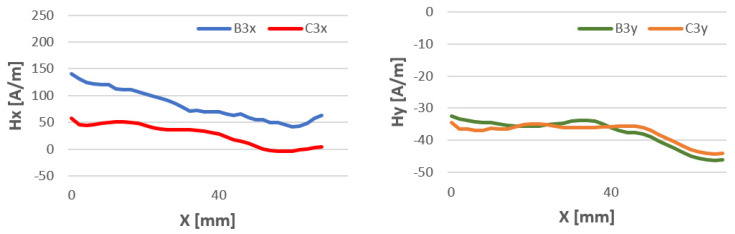
Comparison of the magnetic field distribution Hx and Hy of the binder at temperatures of 80 °C (B) and 15 °C (C)—method 135 MAG.

The study of magnetic field strength in the length of the welded stitch of the test specimens was performed to confirm previous studies. The length of the tested seam l = 70 mm is due to the overall dimensions of the welded sheets. For all welding methods, the Hx and Hy curves have a similar character. The data are shown in Figure 15, Figure 16 and Figure 17.

## 4. Discussion

The purpose of the study was to determine the effect of the welding method on the change in SMLF of welded components. They wanted to investigate whether the welding operation itself causes a change in the magnetic field across the welded component. Additionally, a significant study was conducted to evaluate any modifications in the SMLF of the parent material and the weld during the cooling process. To achieve this aim, the S235JR sheets were prepared, followed by the selection of three welding methods—MMA, TIG, and MAG—and the choice of welding materials in accordance with current standards. By studying the chemical composition of the weld material, it can be concluded that the chemical elements contained in the filler material play a role in changing the magnetic field.

Sulfur present in the welding wire used in the MAG method can disrupt the regular arrangement of magnetic domain structures in ferromagnetic materials such as steel. This disruption can lead to a weakening of the material’s the magnetic flux leakage and a reduction in the magnetization of the welded parts. Therefore, we notice a reduction in magnetic field strength relative to non-welded samples. The high proportion of Mn can result in the material’s ability to maintain its magnetic field in the presence of internal and external magnetic interference. This is evident in tests for TIG-welded wafers. The modification of H(x,y) graphs is contingent on the magnetic field produced during welding, which is method-dependent.

In the MMA method of welding with a covered electrode, electric current flows through the electrode, creating an electric arc between the electrode and the material to be welded. Under the influence of this current, an electromagnetic field is generated around the welding arc. The magnetic field is mainly generated by the electric current and is concentrated around the welding area. Nevertheless, we notice a strong effect on the entire sample (Figure 9). The magnetic field of the sample has been altered along its entire length for both the tangential and normal components. In some places, the field changes by as much as 450 A/mm for the tangential “x” component. At the point where the sheets are joined, the Hx values are similar. For the normal component “y”, we notice a change in the sign of the magnetic field along the entire length tested. 

In the TIG welding process, an electric current flows through an electrode made of tungsten and the welded material. This causes a circular magnetic field around the welding arc. However, the magnetic field generated during TIG welding is usually weak enough to have no significant effect on the welding process. Upon analyzing the data presented in Figure 10, it can be observed that changes have occurred; however, they are comparably smaller than those observed in MMA and MAG welded samples.

During MAG welding, the magnetic field is mainly generated by the flow of electric current passing through the material being welded and the electrode wire. This results in the generation of an electromagnetic field around the welding area.

With sheets welded by this method, we note (Figure 11) that in the graph of Hx values, the magnetic field changes sign from positive to negative after welding. Hy, on the other hand, changes its magnetic properties at the extremities of the tested sheet after the MAG process.

An important part of the study is to obtain information about the changes in the magnetic leakage field in the welded component during the cooling of the weld. Comparing the results of H(x,y) tests (Figure 11, Figure 12 and Figure 13) for different weld temperatures, we notice very small changes in the value of the magnetic field strength. This may be due to the natural cooling process of the weld after welding. The gradual cooling of the steel may lead to the relaxation of stresses caused by the sudden heating and contraction of the weld.

Analyzing the results of H(x,y) measurements, we note that the tangential component of the magnetic field strength, for all methods and materials, takes on positive values. The magnetic field strength for the normal components has only negative values.

Table 4 shows the difference between magnetic field strength measurements at 80^o^ and 15 °C for all repetitions. The arithmetic mean, standard deviation, and measurement limit error were calculated.

(2) $$q_{k}={H(x,y)}_{80\;{}^{\circ}C}-{H(x,y)}_{15\;{}^{\circ}C}$$

Formulas (3)–(5) were used to calculate the arithmetic mean, standard deviation measurement limit error. These formulas and designations are from the Guide to the Expression of Uncertainty in Measurement.

(3) $$\bar{q}=\frac{1}{n}\sum_{k=1}^{n}q_{u}$$

(4) $$S\left(\bar{q}\right)=\sqrt{\frac{1}{n}\sum_{k=1}^{n}{(q}_{k}-\bar{q}{)}^{2}}$$

(5) $$\Delta x=S\left(\bar{q}\right)*k$$

where: $q_{k}$ $random\;variable,\;\bar{q}$—arithmetic mean, *n*—number of repetitions, *S*($\bar{q}$)—the standard deviation Δ*x*—measurement limit error, *k*—the coefficient of expansion, which depends on the assumed confidence level (for a standard 95% confidence level, *k* is about 1.96).

## 5. Conclusions

The purpose of the study was to determine the effect of the welding method and the weld cooling process on the change in the magnetic field strength of S235RJ sheet materials. Based on the results, it can be concluded that:

1. The welding method affects the change of magnetic field strength in the welded material. During the welding process, a magnetic field can occur as a result of the flow of electric current through the welded material and the electrode. The MAG method has the greatest effect on the change in H(x,y) of the methods studied. This is related to the current required for the proper welding of the material.
2. The cooling process that occurs after welding has very little influence on the magnetic field of the welded material, or the weld. The minimal changes that occur are due to the relaxation of thermal stresses.
3. The magnetic field of the weld for the values of the tangential components is always positive, while for the normal component, it has a negative value regardless of the weld material and welding method.

In the future, a test is planned to help determine the residual stress state and detect possible internal defects in welds. For this purpose, ultrasonic (UT) and eddy current (ET) methods will be used using changes in the impedance of an electromagnetic coil eddy current probes can detect very small stress changes in ferromagnetic steels using the magnetoresistive effect based on the measurement of impedance changes [32,33]. A comparison of the residual stresses tested can provide early indications of the stress state and possible failure. The mechanical properties of the welds will be assessed through the use of tensile strength and impact tests. These tests will be held to verify the thesis that the level of magnetic field strength can indicate the quality of the weld.

## Figures and Tables

**Table 1:** Welding process parameters.

Welding Process (by EN ISO 4063:2011 [25])	111 MMA	141 TIG	135 MAG
Joint type	BW (butt weld)	BW (butt weld)	BW (butt weld)
Welding position (by EN ISO 6947 [26])	PA (downhand position)	PA (downhand position)	PA (downhand position)
Base material	S235JR	S235JR	S235JR
Dimension of testing plates [mm]	5 × 70 × 300	5 × 70 × 300	5 × 70 × 300
Filler material	Ø 2.5 mm EB 150	Ø 2.4 mm OK Tigrod 12.64	Ø 1.2 mm wire lincoln
Arc Voltage U [V]	24.4	-	24.2
Welding Current I [A]	98	220	206–211

PA—downhand position—this is the most comfortable position for making any weld (whether using MMA welders, MIG/MAG semi-automatic welders or TIG equipment). The sub-lower position involves applying the electrode from above, preferably perpendicular to the welding surface. This position can be used on flat surfaces laid parallel to the ground, making the process very efficient. It is also a natural body position for the welder.

**Table 2:** Chemical composition and mechanical properties of the filler metal.

Supplementary Material	C	Si	Mn	P	S	Mechanical Properties			
						Rm [MPa]	Re [MPa]	$A_{5}$ [%]	KV (J)
Alkaline electrode—EB 150 ESAB brand	0.08	0.40	1.10	---	---	500–640	>420	>20	>47
OK Tigrod 12.64 ESAB brand	0.10	1.0	1.70	---	---	595	525	26	70
Ø 1.2 mm wire Lincoln brand	0.05	0.40	1.3	0.015	0.010	630	580	24	20 °C = 80 −40 °C = 40

**Table 3:** Weld temperature during cooling.

Welding Process	Temperature Measuring Point	Weld Temperature in °C after			
		Welding	2 Min	5 Min	10 Min
111 MMA	1	581	264	88	15
	2	531	262	83.3	15
	3	594	262	83.3	15
135 MAG	4	487	270	87.7	15
	5	487	270	88	15
	6	534	284	86	15
141 TIG	7	411	185	80.2	15
	8	397	184	80.6	15
	9	407	185	80.6	15

**Table 4:** The average change in H(x,y) after cooling the weld from 80 °C to 15 °C.

Welding Method	Sample Number	$q_{k}$		$\bar{q}$		$S(\bar{q})$		Δx	
		**H(x)**	**H(y)**	**H(x)**	**H(y)**	**H(x)**	**H(y)**	**H(x)**	**H(y)**
111 MMA	1	38.88	11.21	44.9	13.26	5.32	2.89	10.42	5.66
	2	46.87	16.38						
	3	48.96	15.3						
135 TIG	4	43.25	6.87	42.44	7.15	1.14	0.49	2.24	0.97
	5	41.63	7.72						
	6	32.45	6.85						
141 MAG	7	57.54	4.95	52.74	4.65	5.10	0.95	1.86	3.65
	8	53.30	5.42						
	9	47.38	3.59						

## Data Availability

The data presented in this study are available on request from the corresponding author.
